# Supplementary material for: Glaucoma Cases Following SARS-CoV-2 Vaccination: A VAERS Database Analysis
Source: Vaccines (Basel). 2022 Sep 28;10(10):1630. doi: 10.3390/vaccines10101630 (PMC9610013; doi:10.3390/vaccines10101630)
Supplement: Supplementary file 1 [file vaccines-10-01630-s001.zip › vaccines-1921783-supplementary.pdf]

Supplementary Table S1. The country wise distribution of glaucoma cases reported to the CDC VAERS.*

	Vaccine		
	ad26.cov2.s	BNT162b2	mRNA-1273
	Count	Count	Count
**Australia**	0	1	0
**Austria**	0	1	0
**Belgium**	1	6	0
**Czech Republic**	0	3	0
**Denmark**	0	0	1
**Estonia**	0	4	0
**Finland**	0	2	0
**Foreign (non-specific)**	0	1	2
**France**	1	18	1
**Germany**	1	10	1
**Great Britain**	0	12	0
**Greece**	0	2	0
**Ireland**	0	1	0
**Italy**	0	8	1
**Japan**	0	13	0
**Latvia**	0	1	0
**Lithuania**	0	1	0
**Netherlands**	0	3	1
**Norway**	0	0	2
**Philippines**	0	1	0
**Poland**	0	1	0
**Portugal**	0	2	0
**Sweden**	0	1	0
**Taiwan**	0	2	2
**Unknown**	0	3	2
**USA**	1	33	14

* The state-wise distribution of the cases in the United States was included in the VAERS database. The origin of a report from foreign (non-US) countries was identified on the basis of unique identification number assigned by the reporting regulatory body or vaccine manufacturer.

Supplementary Table S2. State-wise crude reporting rate of glaucoma per million doses of COVID-19 vaccinations

	**Glaucoma cases per million doses***		
	**BNT162b2**	**mRNA-1273**	**Ad26.COV2.S**
**Alabama**	0.00	0.37	0.00
**Arizona**	0.29	0.00	0.00
**California**	0.09	0.00	0.43
**Colorado**	0.32	0.00	0.00
**Georgia**	0.00	0.34	0.00
**Illinois**	0.07	0.25	0.00
**Kentucky**	0.28	0.37	0.00
**Maryland**	0.14	0.00	0.00
**Massachusetts**	0.12	0.00	0.00
**Michigan**	0.00	0.16	0.00
**Mississippi**	0.45	0.00	0.00
**Missouri**	0.18	0.31	0.00
**Montana**	1.17	0.00	0.00
**New Jersey**	0.20	0.00	0.00
**New York**	0.04	0.00	0.00
**North Carolina**	0.30	0.00	0.00
**North Dakota**	1.60	0.00	0.00
**Ohio**	0.19	0.00	0.00
**Pennsylvania**	0.38	0.00	0.00
**South Carolina**	0.23	0.00	0.00
**Tennessee**	0.00	0.26	0.00
**Texas**	0.04	0.00	0.00
**Virginia**	0.00	0.34	0.00
**Washington**	0.00	0.18	0.00
**West Virginia**	0.68	0.85	0.00
**Wisconsin**	0.33	0.26	0.00

*Vaccine data from Centers for Disease Control and Prevention on April 30, 2022

Supplementary Table S3. Therapeutic interventions reported in patients diagnosed with glaucoma post-COVID-19 vaccination

	**Frequency**	**%**
Brimonidine	5	3.1
Dorzolamide	6	3.7
Eye drops (not specified)	18	11.2
Laser iridotomy	18	11.2
Mannitol	2	1.2
Shunt/Valve placement	4	2.5
Surgery (not specified)	7	4.3
Timolol	11	6.8
Trabeculectomy	2	1.2
Travoprost/Latanoprost	2	1.2
No/unknown intervention	86	53.4
